# Monthly Summary

**Published:** 1897-07

**Authors:** 


### Monthly Summary.

NEW METHOD OF ADMINISTERING CHLOROFORM.—Dr. Rosenbery asserts that the disturbances of the action of the heart and of respiration in administering chloroform, and also with other anæsthetics, is caused by irritation of the nerve branches of the pituitary mucous membrane, and attempts have been made to prevent this by benumbing the membrane with cocaine. After a test of fifty cases Dr. R. gives the following advantages of this method: 1. The first stage of anæsthesia is less unpleasant, and the patient never struggles against inhaling the chloroform. 2. The stage of excitement rarely occurs, and when it does it is very slight, except in the case of hard drinkers. 3. Only in a very few cases does vomiting occur, and then preceded by only slight nausea. 4. After recovery no unpleasant sensation is experienced. The cocaine is applied as follows: The patient is directed to blow the nose so as to thoroughly free it of mucous. Then he is placed into a sitting position, rather leaning forwards (never in a lying position) and made to snuff one centigramme of powder composed of a mixture of 10 per cent. bidrochlorate cocaine with an indifferent powder. After about three minutes repeat, and immediately commence the chloroform.—*Correspondenz Blatt.*

[I have used a cocaine spray in the nose for the same purpose.—C. E. Klotz.]

---

PEARSON'S LOCAL ANÆSTHETIC is composed of the following:

| | |
|---|---|
| Chloroform, | 12 |
| Tinct. Aconite, | 12 |
| " Capsici, | 4 |
| " Pyrethri, | 2 |
| Ol. Caryophylli, | 2 |
| Camphor, | 2 |

The camphor is dissolved in the chloroform, the Ol. Caryophylli added, and then the tinctures.—*Zahuarztliches Wochenblatt.*

PULP DEVITALIZATION IN THE TEETH OF CHILDREN.—By Dr. Darby.—One of our great difficulties in dealing with the teeth of children is the devitalization of the pulp when indicated. I have used, and with much success for this purpose, a paste of powdered cantharides and carbolic acid; say about one-twentieth grain of the powder with enough carbolic acid or creosote to make a paste. I know that the use of arsenic for this purpose is justly viewed with suspicion, but my opinion is that it is largely a question of how much arsenic is used. I use arsenic for this purpose in very minute quantities and have had no ill results. The canals of children's teeth should, of course, be cleansed thoroughly and sterilized. I question the use of cotton dressing in these cases, for should the foramen be large, owing to a partial resorption of roots, soft tissues might be impinged upon, and the cotton becomes a source of irritation or worse. I think the safer practice is to use fluid in the canals and oxychloride in the pulp chamber.—*International.*

---

A LITTER OF SUPERNUMERARY TEETH.—By G. Lenox Curtis, M. D.—A patient aged 21 years, with a history of a full set of deciduous teeth and no extraction of the permanent set, presented herself, complaining of pain and some swelling in the jaw near the symphysis. Examination revealed the absence of the inferior left lateral incisor, the left central being slightly loose and tender on pressure. A slight space intervened between this and the cuspid, the cusp of which was tilted forward and the apex well back, encroaching upon the bicuspid. Suspecting an unirrupted lateral, a shell probe was passed through the gum and periosteum until a glistering subject resembling enamel was reached. The patient was so highly sensitive and hysterial that further examination was deferred until the time of operation. An incision in the gum was made directly over the tumor at the apex of the root, the periosteum was opened and laid aside so as to expose the contents. Here were found seven teeth nestling together in a cavity about the size of a denuded peanut. The roots were like the spokes of a wheel radiating outwards, some almost penetrating the gingival margin of the gum projecting above, while others were close to the lower border of the jaw. The cavity in which they were confined was perfectly smooth and without a soc. The roots of the teeth were pointed like tacks and were from one-sixteenth to a quarter of an inch in length. The crowns uniformly being an eighth. They closely resembled small inferior incisors of the temporary set. The crowns of only two showing clear enamel, the others being clump shape with a growth of yellowish color much resembling exostosis. On opening the teeth I found they contained a pulp same as a normal tooth. This beats my record by four at a single birth. Around one of the largest teeth was a soc highly inflamed filled with fluid, and from this, no doubt, the trouble arose.—*Dominion Dental Journal.*

---

METAL PINS for use in the roots of pulpless teeth as anchorage posts may be made of platinum alloyed with iridium, gold alloyed with platinum or of stiff German silver wire, the latter being, of course, much less expensive but not quite as reliable for the demands in all respects as the two first named. This wire in several different sizes (say Nos. 3 to 7 of bur-gauge) should always be at hand, and before being used a shallow screw thread should be cut upon it merely for the purpose of giving proper hold to whatever plastic material may be used in connection with it. The writer prefers to select a screw a trifle smaller than the calibre of the prepared root-canal, and cement it in place with zinc phosphate, rather than use a screw slightly larger than the canal, depending upon the thread cut in the dentine by screwing it in as some operators do. What has been said above against the use of zinc phosphate refers, of course, only to its use in locations reached by the saliva. Stiff German silver is not as rigid as the other wires referred to, but will always answer if two roots can be utilized, a pin being placed in each. It is not affected by mercury, nor is ordinary clasp metal—gold and platinum—by the proportion of mercury in amalgam, though pure mercury will sometimes affect it slightly. Still German silver has been referred to because there are two varieties of the metal obtainable. The largest round bar used in preparing the root canal for the reception of the metal pin should be measured in the bur-gauge, and a pin just one size smaller selected for use.—S. E. Davenport, D. D. S., M. D. S., in *N. Y. International Dental Journal.*

---

TREATING PULPLESS TEETH.—What does scientific treatment really consist of? As I understand it, the crowning point of all scientific treatment is the result. If the result is favorable, than we want nothing in the way of science than that, so far as treatment is concerned. You may say that there may be germs and spores left here and there, but supposing they are put in such a condition that they are unable to accomplish any harm. I think there is hardly anything ever put into the roots of teeth, or into the canalicula of the dentin, that will always remain aseptic, or keep its parts absolutely in that condition. Still we have teeth that have been going on for twenty-five and thirty years, and with the modern treatment we have had teeth going on for ten and twelve years without the slightest disturbance. Do we want anything better than that?

I wish to call attention to the treatment of roots of teeth which I have advocated and which I have practiced for at least ten years; and I have yet to see, I am almost ready to say, one single failure in that treatment. They are so few that they are hardly worth mentioning. The treatment in short is as follows: the moment the tooth is opened the canal and contents are put under the influence of a powerful antiseptic, and kept that way from that time on. It is not allowed again to become septic. Before the patient leaves my chair the root and tooth are filled. I do not dry the canal, but carry the oxichloride cement into the antiseptic, with which it mixes, and the mass crystallizes, thus forming a permanent filling. After thus filling the canal, the crown is filled with gold or other material as I choose. Gentlemen of long experience will agree with me when I say it is almost an impossibility to dry canals of many teeth in the mouth. Many have gone so far as to attempt to dry them with hot air, with I believe not the best results. Not only has hot air been used to dry pulp-canals, but with the expectation and avowed purpose of drying the substance of the dentin itself, the claim being that by so doing the canals and canalicula were thoroughly sterilized. Is this ever accomplished? Is there anything in it that commends itself to a man of experience in the treatment of pulpless teeth? I think not.—Prof. Frank Abbott, in American Dental Association.—*Welch's Monthly.*

---

CATCHING COLD.—The "cold spots," meaning the surface areas peculiarly susceptible to cold, are principally the nape of the neck and the lower part of the back of the head, the front of the abdomen and the shins. The acute discomfort and the sense of impending disaster which results from the steady play of a current of cold air on the neck from behind are well known. The necessity of keeping the abdomen warmly clad is also generally recognized, though perhaps not as generally carried into practice. Curiously enough, few people are conscious of the danger they run by exposing the usually inadequately protected shins to currents of cold air. This is usually the way in which colds are caught on omnibuses. When driving we take care to cover the legs with a rug or water-proof, but on the more democratic conveyance rugs are not often available, and the reckless passenger by-and-by awakens to the fact that the iron has entered into his soul—in other words, that he has "caught cold." People who wear stockings, such as Highlanders, golfers and cyclists, invariably take the precaution of turning the thick woolen material down over the shins, the better to protect them against loss of heat, though, incidentally, the artificial embellishment of the calves may be altogether foreign to the maneuver. This is an instance of how all things work together for good. It does not, of course, follow, because some areas are peculiarly susceptible to cold, that a chill may not be conveyed to the nervous system from other points. Prolonged sitting on a stone, or even on the damp grass, is well known to be a fertile source of disease; and wet cold feet are also, with reason, credited with paving the way to an early grave. *London Medical Press.*

THE SENSE OF TOUCH.—Of all the senses, touch is the most exquisite and reliable, and the most susceptible to improvement. And of all parts of the body the finger tips are the most wonderfully sensitive and discriminating in carrying impressions to the brain. In some trades experts in this faculty are paid high salaries for their services. The solidity, fineness, or sharpness of grain, the surface, texture, or ingredients of mixtures or solids, and the value of minerals, earths or other materials of nature or manufacture, are largely ascertained by the touch. The blind are notable instances of the marvelous degree of cultivation to which this may be carried. Its promptness, precision and practical value is astonishing. The fingers of the dentist are wonderfully educated in this direction. With remarkable accuracy he judges of the condition, structure and varied phases of healthy or diseased tooth substance, though he only feels it through the point of an instrument. The physician judges of the condition of his patient largely by the sense of touch. Even hidden structures are thus diagnosed. It is by the high cultivation of this sense of touch that the painter, musician and artisan attains distinction. No other sense gives pleasure or pain; for it is only to slightly increase a touch or pressure that gives pleasure, to have the sensation one of pain. In fact, the only cause of physical pain is undue pressure on the nerves, and the only source of physical pleasure is the thrill of the touch that is agreeable.— *Welch's Monthly.*

---

THE ANTISEPTIC PROPERTIES OF SALIVA.—Hugenschmidt (*Ann de l' Inst. Pasteur*, October, 1896), has made an attempt to determine why operations on the mouth are so seldom followed by infection. He was not able to prove that the saliva possesses any permicidal power. The more common bacteria grow rapidly in it. Nevertheless, it has a twofold mechanical action to keep down infection. It dilutes and washes away alimentary material, and by its alkalinity it prevents fermentation.

But Hugenschmidt thinks the saliva possesses the power of stimulating diapedesis in the numerous lymphatic organs situated in the mouth. Even in its normal state the saliva contains products of the secretion of microbes, and thus favors the phagocytic action.

This hypothesis the writer upholds by several experiments. One of them was the introduction of small glass tubes into the abdomen of the guinea pig. These tubes were closed at one end and filled with saliva. In eight hours it was found that the leucocytes had crowded into the open end of the tube, forming a plug about two mm. in thickness.

In a second experiment, a small portion of the mucous membrane of the lower jaw of the guinea-pig was resected and its base mangled with a curette. In twenty-four hours the wound was covered with a layer of leucocytes, in the interior of which were found bacteria of various sorts, apparently of the same sorts as those in the saliva. From these, and other experiments, Hugenschmidt concludes that the saliva has no germicidal power, and that the freedom from infection of wounds in the mouth is due to active phagocytosis, brought about by the power of the saliva to stimulate the diapedesis of leucocytes.—*Medical News.*

---

A USEFUL FLUX.—A flux that is exceedingly useful in bridge-work is prepared as follows:

Put in a cup—

| | |
|---|---|
| Boracic acid | 1 oz. |
| Ammonia | ½ oz. |
| Carbonate of ammonia | ½ dwt. |
| Bicarbonate of soda | 2 dwt. |
| Water | 4 oz. |

Boil till the fumes of ammonia are no longer given off.

Coat the bridge or other work all over the gold with the flux. Heat it over a spirit lamp to dry it on. Give it another coat, if needed, leaving no part exposed. Then scrape off where it is desired that the solder shall flow, and it will go nowhere else. The work will come out of the heating as bright as when it went in, and the solder will be smooth. The polished surfaces will not be corroded or blackened.—*Western Dental Journal.*

